# Abnormal Metabolism in the Progression of Nonalcoholic Fatty Liver Disease to Hepatocellular Carcinoma: Mechanistic Insights to Chemoprevention

**DOI:** 10.3390/cancers13143473

**Published:** 2021-07-11

**Authors:** Danny Orabi, Nathan A. Berger, J. Mark Brown

**Affiliations:** 1 Department of Cardiovascular and Metabolic Sciences, Lerner Research Institute of the Cleveland Clinic, Cleveland, OH 44106, USA; orabid@ccf.org; 2 Center for Microbiome and Human Health, Lerner Research Institute of the Cleveland Clinic, Cleveland, OH 44106, USA; 3 Department of Molecular Medicine, Cleveland Clinic Lerner College of Medicine of Case Western Reserve University, Cleveland, OH 44195, USA; 4 Case Comprehensive Cancer Center, Cleveland, OH 44106, USA; nab@case.edu; 5 Department of General Surgery, Cleveland Clinic, Cleveland, OH 44195, USA; 6 Department of Medicine, Case Western Reserve University School of Medicine, Cleveland, OH 44106, USA; 7 Department of Biochemistry, Case Western Reserve University School of Medicine, Cleveland, OH 44106, USA; 8 Department of Genetics and Genome Sciences, Case Western Reserve University School of Medicine, Cleveland, OH 44106, USA

**Keywords:** nonalcoholic fatty liver disease, nonalcoholic steatohepatitis, hepatocellular carcinoma, PPAR, bile acids, gut microbiome, autophagy

## Abstract

**Simple Summary:**

Nonalcoholic fatty liver disease (NAFLD) is a disorder of excess lipid accumulation within the liver. Rates of NAFLD have rapidly increased with the sudden rise in obesity and metabolic syndrome. NAFLD may lead to liver inflammation, fibrosis, and dysfunction, and in select cases, the development of liver cancer in the form of hepatocellular carcinoma (HCC). NAFLD is established as a leading cause of liver cancer, and its contribution is expected to grow. Abnormal hepatic lipid metabolism has been repeatedly associated with the progression of NAFLD to primary liver cancer. The current treatment of NAFLD is focused primarily on reducing liver inflammation and fibrosis. Using therapies that additionally target metabolic or lipid signaling pathways that are responsible for tumor development may work to reduce the risk of liver cancer in these patients.

**Abstract:**

Nonalcoholic fatty liver disease (NAFLD) is on the rise and becoming a major contributor to the development of hepatocellular carcinoma (HCC). Reasons for this include the rise in obesity and metabolic syndrome in contrast to the marked advances in prevention and treatment strategies of viral HCC. These shifts are expected to rapidly propel this trend even further in the coming decades, with NAFLD on course to become the leading etiology of end-stage liver disease and HCC. No Food and Drug Administration (FDA)-approved medications are currently available for the treatment of NAFLD, and advances are desperately needed. Numerous medications with varying mechanisms of action targeting liver steatosis and fibrosis are being investigated including peroxisome proliferator-activated receptor (PPAR) agonists and farnesoid X receptor (FXR) agonists. Additionally, drugs targeting components of metabolic syndrome, such as antihyperglycemics, have been found to affect NAFLD progression and are now being considered in the treatment of these patients. As NAFLD drug discovery continues, special attention should be given to their relationship to HCC. Several mechanisms in the pathogenesis of NAFLD have been implicated in hepatocarcinogenesis, and therapies aimed at NAFLD may additionally harbor independent antitumorigenic potential. This approach may provide novel prevention and treatment strategies.

## 1. Introduction

In the United States, primary liver cancer ranks fifth in cancer-related mortality [1], with hepatocellular carcinoma (HCC) comprising the majority of these tumors [2]. Nonalcoholic fatty liver disease (NAFLD) has recently emerged as a leading etiology [3], and some predict NAFLD will become the predominant cause of HCC in the next decade. This is in part due to advancement of vaccination programs and direct-acting antiviral therapies contributing to a decrease in chronic viral hepatitis cases, coupled with the continuing rise in obesity in developing nations [4,5]. While obesity is associated with a striking 40% of all cancers in the United States [6], the contribution of obesity and aberrant metabolism to hepatic malignancies is particularly important because of the central role that the liver plays in the metabolism of lipids and other nutrients. Several metabolic pathways contributing to the pathogenesis of NAFLD have been implicated in HCC development and progression, which may serve as novel therapeutic targets for prevention and treatment.

The American Association for the Study of Liver Diseases (AASLD) defines NAFLD as greater than five-percent liver steatosis in the absence of secondary causes such as alcohol or medications [7]. This may be further classified as liver steatosis in the absence or presence of liver inflammation and designated as nonalcoholic fatty liver (NAFL) or the more advanced manifestation of nonalcoholic steatohepatitis (NASH), respectively. The chronic inflammation produced by NASH may promote hepatic collagen deposition and fibrosis [8]. If severe enough, this leads to cirrhosis and critical liver dysfunction (i.e., end-stage liver disease). When HCC formation occurs in a patient with a history or histological pattern of NAFLD in the absence of viral, alcoholic, or other liver disease history, the etiology of HCC is presumed to be from NAFLD and will be referred to as NAFLD-related HCC in this text.

There are currently no Food and Drug Administration (FDA)-approved medications for the treatment of NAFLD. Additionally, while rates of NAFLD-related HCC are on the rise [3,4,5], no medications are currently recommended for the treatment of NAFLD as a primary preventive approach against HCC. Rather, the current efforts aimed at NAFLD-related HCC are focused on tools that improve screening, enable early detection, and inform timely effective intervention [7,9]. The treatment of NAFLD-related HCC is not distinguished from non-NAFLD cases despite a distinct pathogenesis and genetic landscape [10,11]. Systemic therapies for HCC have evolved from conventional cytotoxic chemotherapy to multikinase and checkpoint inhibitors, but 5-year survival still remains below 20% and improved prevention strategies are necessary [1]. Adopting an approach focused on reducing HCC development in NAFLD patients through the modulation of key metabolic pathways responsible for tumor development should provide valuable chemoprevention.

The association between aberrant metabolism and liver disease has traditionally been known as NAFLD. However, the widespread prevalence of metabolic dysfunction and the limitations imposed by the current definition of NAFLD have led an international consensus panel to propose a revised classification named metabolic-associated fatty liver disease (MAFLD) [12]. This revised classification advocates for diagnosis based primarily on the presence of metabolic dysfunction rather than the absence of secondary causes of liver disease, thereby making the diagnosis one of inclusion rather than exclusion. This revision is significant because it recognizes the often-present metabolic dysfunction that exacerbates liver pathology in chronic liver disease of various etiology, especially alcoholic liver disease. Emphasis on these metabolically focused pathogenic mechanisms will help to better understand disease progression and guide treatment. Since NAFLD is the traditionally designated form of disease, its definition will be used herein. However, we recognize that MAFLD will likely become the new designation given its more accurate and comprehensive approach.

## 2. Epidemiology and Predictions

### 2.1. NAFLD

A recent meta-analysis by Younossi et al. that limited inclusion criteria to studies in which diagnosis was based on imaging reported a 25% prevalence of NAFLD in the United States [13]. While rates showed significant regional variation, a similar 25% prevalence was found globally. The prevalence of NASH among American NAFLD patients with or without an indication for liver biopsy was estimated to be 60% and 30%, respectively. Highest rates of NAFLD are seen in Hispanics followed by Americans of European then African descent [14]. Initially, gender differences in NAFLD were unclear, with some reporting an increased prevalence among females [14], though recent data actually suggest a higher prevalence and severity among men compared to premenopausal women, with estrogen conferring a protective effect [15]. While studies may vary in reported prevalence of NAFLD due to differences in study populations and diagnostic criteria, there is little debate that rates are rising both in the United States and worldwide [13,14,16,17,18]. Cryptogenic cirrhosis is now being recognized as a form of “burnt-out NASH”, which describes the loss of steatosis in NASH patients with advanced fibrosis, and may account for the historical underestimation of disease burden [4,14,19].

### 2.2. NAFLD-Related HCC

A review detailing the epidemiology of NAFLD-related HCC by Kogiso et al. is included in this special edition of MDPI Cancers [20]. Here, we will briefly summarize key epidemiologic concepts and predictions relevant to this review.

Leading etiologies of HCC in the United States and worldwide are chronic infection with hepatitis B or C virus, chronic excessive alcohol consumption, and NAFLD, with the genetic (e.g., alpha 1 antitrypsin, hemochromatosis) and environmental (e.g., aflatoxin) causes being less frequent [3,4,5]. Current estimates report NAFLD-related HCC to constitute 10–20% of HCC in the United States [3,4,21,22], though variability in definitions, diagnostic criteria, and patient populations confound these epidemiologic studies. Older age, male sex, Hispanic ethnicity, and the presence of cirrhosis are risk factors for HCC development in NAFLD patients [23]. NALFD is the fastest growing indication for HCC-related liver transplantation in the United States, and currently ranks second only to hepatitis C infection [24]. While the risk estimate of HCC development in NAFLD patients is approximately 10-fold lower than that of hepatitis B or C infection, the overwhelming prevalence of NAFLD has propelled it to become a leading cause [4,22]. Strong advancements in hepatitis B and C virus prevention strategies and treatment will continue to lessen their attributable HCC burden [4,5].

Cryptogenic cirrhosis, representing “burnt-out NASH”, has led studies to attribute HCC etiology in such cases to NAFLD [4,14,19]. A variation of this requires HCC patients with cryptogenic cirrhosis to additionally have at least one component of metabolic syndrome (obesity, diabetes, dyslipidemia, or hypertension). The rationale for this specification is that metabolic syndrome increases the likelihood of concurrent NAFLD and that these metabolic derangements independently confer higher risk of HCC development. The contributions of individual components of metabolic syndrome on HCC development and progression will be highlighted in the next section.

## 3. HCC and Metabolic Syndrome

### 3.1. Metabolic Syndrome

**Figure 1:**
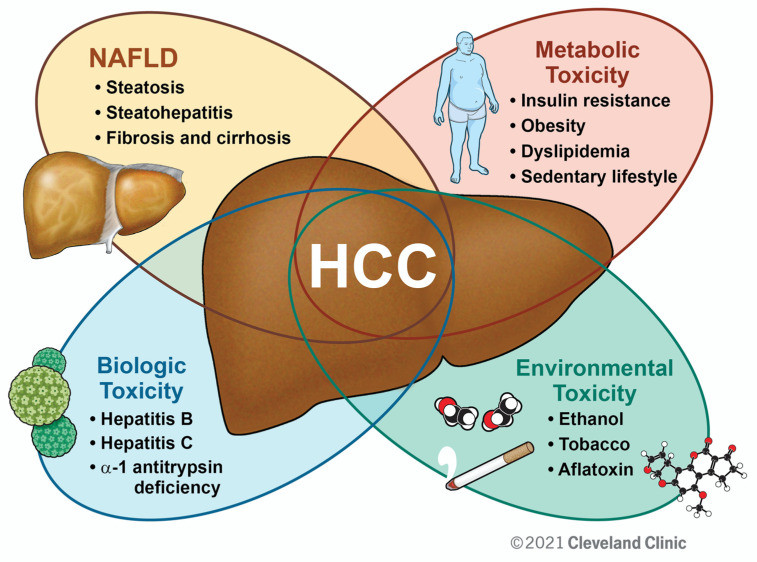
The Role of NAFLD and Metabolic Syndrome in HCC. Illustrated is a working model by which diverse factors may compound one another to promote HCC. While individual patients will have certain “drivers” of disease, emphasis should be placed on improving metabolic parameters as a primary preventive strategy against HCC from chronic liver disease of various etiologies.

Metabolic syndrome, or “syndrome X,” describes the constellation of obesity, hyperglycemia/insulin resistance, dyslipidemia, and hypertension [25,26]. It has now become accepted that NAFLD is both a cause and consequence of metabolic syndrome through the interrelationship of liver metabolism and systemic metabolic homeostasis [27,28]. There is a tendency to view metabolic syndrome and NAFLD as a separate, irrelevant entity in the context of non-NAFLD-related HCC. However, metabolic derangements and NAFLD confer higher HCC risk and poorer prognosis in patients with chronic liver disease of various etiologies and will be discussed below. We propose HCC as the culmination of a complex multifactorial process with metabolic syndrome and NAFLD playing key roles (Figure 1). An emphasis should be placed on improving overall metabolic health in all patients with chronic liver disease, especially those at high risk for HCC development.

### 3.2. Obesity and Dyslipidemia

In addition to lipid storage, adipose tissue functions as an endocrine organ modulating hormones and cytokines to affect insulin resistance, endothelial dysfunction, systemic inflammation, and much more [29,30]. Rising rates of NAFLD have been accompanied by the rise in obesity over the last half century [31,32]. NAFLD risk markedly increases with higher body mass index (BMI) in a dose-dependent manner, with obesity (BMI > 30 kg/m^2^) conferring a 3- to 7-fold higher risk [33,34]. Obesity additionally serves as an independent risk factor for HCC overall [35,36,37], as well as in hepatitis C and alcoholic cirrhosis [38,39,40], but does not appear to do so in chronic hepatitis B infection [38,41]. The effect of obesity on prognosis and survival is confounded by differences in stage at diagnosis, treatment modalities, and gender differences, as well as by overall patient decompensation secondary to advanced tumor burden and treatment effects [42]. The use of obesogenic high-fat diets in animal models of HCC supports its pro-inflammatory and tumorigenic effect [43,44]. In addition to fostering HCC development indirectly by promoting NAFLD progression, obesity may independently promote HCC development through adipokine-mediated immunomodulation and carcinogenesis [45].

Dyslipidemia and notably hypertriglyceridemia are key components of metabolic syndrome. The relationship between dyslipidemia, NAFLD, and HCC is obscured by the liver’s role in lipid homeostasis. While elevated cholesterol and triglyceride levels are clear risk factors for NAFLD [46,47], the liver dysfunction frequently seen after HCC development is associated with decreased serum lipid levels [48,49].

### 3.3. Insulin Resistance

Insulin resistance denotes the impaired ability of target tissues to respond to insulin signaling and effectively maintain systemic glucose homeostasis [50]. This leads to hyperglycemia, initially with hyper- followed by hypoinsulinemia from pancreatic beta-cell failure. This directly and indirectly affects hepatic lipid metabolism to promote NAFLD [28,50,51]. Epidemiologic studies support an independent role for diabetes in the development of HCC, regardless of etiology [36,52,53,54]. It should be noted, however, that liver disease may promote the development of diabetes, thereby confounding these population studies, and data should be interpreted with caution. Nonetheless, the tumorigenic role of insulin resistance using in vitro and animal models strongly supports causation. For example, insulin resistance has been shown to promote hepatic steatosis via insulin receptor substrate-1 (IRS1) activation of sterol regulatory element-binding protein-1c (SREBP1c), the major regulator of hepatic fatty acid synthesis [51,55]. Similarly, IRS1 and SREBP1c were found to promote HCC development and progression [56,57].

Metabolic syndrome is intimately related to HCC development and progression in chronic liver disease of various etiology. The pathogenic mechanisms conferring this increased risk are being investigated with an emphasis on their role in the development of NAFLD-related HCC. However, mechanisms found to be responsible may additionally play pathogenic roles in HCC of non-NAFLD etiology. Metabolic parameters should be optimized in NAFLD and non-NAFLD chronic liver disease patients with or without HCC to mitigate the detrimental, tumorigenic effect of metabolic syndrome.

## 4. Clinical Approach to NAFLD and NAFLD-Related HCC

### 4.1. Current Diagnostic and Treatment Strategies of NALFD

The latest AASLD Practice Guidance recommendations will be used to summarize diagnostic and treatment approaches for NAFLD [7]. Due to the lack of evidence, no current screening practices are recommended and NAFLD is most often incidentally diagnosed. Differentiating NAFL from NASH has clinical significance as the latter indicates substantially higher morbidity [13,58,59]. While liver biopsy is the gold standard to diagnose NASH and its severity, it is invasive and costly. There have been advances in non-invasive diagnostic methods, in particular validated predictive score models, serum markers representative of liver fibrosis, and advanced non-invasive imaging techniques [60].

Early treatment of NAFLD aims at lifestyle modification and avoidance of exacerbating factors, for which the literature has provided strong support. Weight loss, exercise, and avoidance of heavy alcohol use have repeatedly been shown to significantly improve markers of disease, even reversing early fibrosis in some cases [61,62,63,64,65]. Pharmacologic therapy in NAFLD is reserved for patients with NASH and often limited to those with advanced fibrosis. Bariatric surgery is considered in NAFLD patients unable to meet weight loss goals and has been shown to impressively reverse NAFLD and decrease the risk of HCC [9,66], further supporting preventative strategies that focus on managing the metabolic disturbances associated with obesity. The major liver-directed drug therapies in use today include pioglitazone, liraglutide, and vitamin E, and these will be discussed in detail in the following sections. Obeticholic acid, a bile acid analogue and farnesoid X receptor (FXR) agonist, is in an ongoing phase 3 clinical trial and elafibranor, a dual peroxisome proliferator-activated receptor (PPAR) α/δ agonist, recently completed its phase 3 trial and will also be discussed in the following sections. Less prevalent medications used specifically for their liver-directed effects are statins and aspirin [67]. Metformin and omega-3 fatty acids are considered though do not appear to provide benefit [68,69].

### 4.2. Current Prevention and Treatment Strategies of NAFLD-Related HCC

The prevention strategy of NAFLD-related HCC parallels the treatment of NAFLD. This stems from the notion that HCC develops more commonly in advanced liver disease, i.e., late-stage fibrosis or cirrhosis, and HCC screening is currently only recommended for NAFLD patients with cirrhosis [7,70]. However, the approach is problematic as NAFLD-related HCC often develops in the absence of cirrhosis [4,23,71,72], though this is unclear and may vary by population [20,70,73]. Nonetheless, patients with NAFLD-related HCC are often diagnosed at a later stage compared to their viral and alcoholic counterparts and advances in screening and prevention are needed [3,72,74].

The treatment approach for NAFLD-related HCC is not distinguished from HCC of different etiology [70]. Curative approaches involve surgical resection or liver transplant, though advanced stage disease at presentation often eliminates these options. Adjuvant treatments include ablation, radio- and chemoembolization, radiation therapy, and systemic therapy. Sorafenib and other related multikinase inhibitors in addition to nivolumab, a checkpoint inhibitor, have emerged as the leading systemic therapies in HCC. Conventional cytotoxic chemotherapy is infrequently used.

## 5. Therapeutic Targets in NAFLD and NAFLD-Related HCC

Pathogenic mechanisms responsible for NAFLD progression may also independently promote HCC. Therefore, therapeutic strategies that primarily prevent NAFLD progression may have strong potential to likewise improve HCC outcomes. Numerous clinical trials have investigated the safety and efficacy of prospective NAFLD therapies with mixed results. Here, we briefly review current molecular targets that have been explored for NAFLD, and discuss how they may hold promise in NAFLD-related HCC.

### 5.1. PPARs

PPARs are nuclear receptors that play intimate roles in glucose and lipid metabolism [75]. Three isoforms exist, each varying in tissue distribution and function. The most well-known is PPARγ, the target of the thiazolidinedione (TZD) class of medications that are in widespread use today. PPARγ is primarily expressed in adipocytes and promotes adipocyte differentiation and lipogenesis. PPARα is highly expressed in liver and functions in fatty acid uptake and beta-oxidation. PPARβ/δ, also known as PPARβ or PPARδ, plays a prominent role in intestinal, liver, and skeletal muscle lipid metabolism, affecting mitochondrial function and fatty acid oxidation. All three isoforms have been established to play a role in NAFLD development and progression [75,76], and have additionally been implicated in HCC.

#### 5.1.1. Thiazolidinediones and PPARγ

PPARγ is highly expressed in adipocytes, where it plays a critical role in the transcriptional control of adipocyte differentiation and triglyceride metabolism [75]. Ligand-dependent activation of PPARγ increases lipogenesis and decreases lipolysis, promoting the accumulation of cellular lipids such as triglycerides [75]. Thiazolidinediones (TZDs) are the predominant class of PPARγ agonists that have been used clinically, and pioglitazone and rosiglitazone are currently available in the United States. TZDs are most known for their insulin-sensitizing ability and their leading role in the treatment of hyperglycemia, though are currently second- or third-line antidiabetic agents due to their adverse effects [77]. TZDs exert their antihyperglycemic effects via PPARy-mediated adipocyte proliferation and lipogenesis to reduce circulating free fatty acids, as well as via modulation of adipokine levels and relevant cytokines [75,78,79]. These effects additionally reduce NAFLD progression [78,79]. Multiple clinical trials have demonstrated reduced steatosis, inflammation, and fibrosis in NAFLD patients with pioglitazone therapy [78,80]. Currently, AASLD treatment guidelines recommend pioglitazone in NASH patients with or without concomitant diabetes, with careful consideration of patient risk versus benefit [7].

The association between prior TZD treatment and lower HCC incidence is well described [52,81,82,83,84,85,86], with a prospective case–control study by Hassan et al. at MD Anderson Cancer Center in Houston, Texas demonstrating up to a 70% risk reduction [52]. Pioglitazone has been shown to exhibit numerous direct antitumorigenic roles in a variety of cancers [87], especially hepatocellular carcinoma [88,89,90]. In a recent study by Li et al., pioglitazone improved fibrosis and HCC tumor burden in two rodent models of HCC [88]. Adiponectin-mediated 5’-adenosine monophosphate (AMP)-activated protein kinase (AMPK) activation with subsequent mitogen-activated protein kinase (MAPK) downregulation was implicated. Several other studies have shown direct antitumorigenic effects via PPARγ activation [90,91]. Interestingly, early literature suggest pioglitazone may also achieve direct antitumorigenic effects through PPARγ-independent mechanisms [92,93], though more investigation is needed. In summary, population studies and preclinical models of disease provide evidence for the use of pioglitazone as chemoprevention of HCC. A limitation of population studies is their retrospective nature as well as heterogeneous cohorts with many non-NAFLD patients. A prospective randomized study is needed.

#### 5.1.2. Fibrates and PPARα

In humans and rodents, PPARα is predominantly expressed in the liver, where it plays a major role in hepatic and systemic lipid metabolism [94,95]. Early PPARα-activating drugs include the fibrates, notably fenofibrate, gemfibrozil, bezafibrate, and clofibrate. Rodent models have consistently demonstrated the role of PPARα in fatty acid transport and beta-oxidation, leading to improvement in serum lipid profiles, hepatic steatosis, and insulin resistance [96,97,98,99,100,101]. Fibrates are currently used for the treatment of dyslipidemia, particularly hypertriglyceridemia. Large clinical trials demonstrated their ability to improve serum lipid profiles with relatively few adverse events [102,103], though side effects include transient hepatic transaminase elevations and myopathy [104,105,106]. The ability of PPARα activation to improve liver steatosis and steatohepatitis as well as insulin resistance in rodents led to a few small clinical studies to determine the effects of fibrates on NAFLD progression [107,108,109,110,111]. Some of these studies demonstrated improvement with fibrate therapy, though data were limited by low sample sizes, the use of combination therapy, and suboptimal methods of assessment. The excitement of PPARα activation in the treatment of NAFLD subsided until recently with the emergence of selective PPARα modulators (SPPARMα), a class of compounds designed to more precisely and potently target PPARα while minimizing side effects. Permafibrate (or K-877), a SPPARMα, was shown to improve dyslipidemia over both placebo and fenofibrate administration in a phase 2 clinical trial [112]. Honda et al. demonstrated improvements in obesity, dyslipidemia, and histological markers of NAFLD in two rodent models of NASH using permafibrate treatment, with increased hepatic expression of acyl-CoA oxidase 1 (ACOX1) and uncoupling protein 3 (UCP3) [113]. Another preclinical rodent model of NASH demonstrated permafibrate’s ability to reduce NASH progression via immune cell alterations [114]. Recent phase 2 and 3 trials demonstrate permafibrate’s ability to treat dyslipidemia and also improve Homeostatic Model Assessment for Insulin Resistance (HOMA-IR) scores in diabetic patients with a low rate of adverse events [115,116]. A recent small study of 10 biopsy-proven NASH patients showed improvements with permafibrate therapy by non-invasive assessments of fibrosis (fibrosis-4 index, aspartate aminotransferase-to-platelet ratio index), further supporting a potential role for PPARα in the treatment of NAFLD [117].

Effects of PPARα activation on HCC have been clouded by the marked interspecies differences in liver PPARα gene expression and target gene modulation between humans and rodents [118]. It was found early on that PPARα activators, fibrates and WY-14643, led to the formation of paraneoplastic foci and HCC in mice and rats after 50 weeks of administration [119,120], though thorough subsequent investigation has found this does not translate to human studies [121,122,123]. PPARα knockout mice were found to be more susceptible to diethylnitrosamine (DEN)-induced HCC with nodules demonstrating a higher proliferative index by Ki-67 staining and decreased levels of apoptosis through knockout-mediated activation of NF-κB signaling [124]. Several in vitro studies on cultured human hepatocytes have demonstrated a direct antitumorigenic role of fibrates mediated via changes in cyclin-dependent kinase (CDK) and cyclin levels as well as apoptotic protein levels along with radical oxygen species (ROS) formation, protein kinase B (AKT) signaling, and fatty acid synthase (FASN)-mediated lipid synthesis [125,126,127,128]. Some of these effects were found to be mediated via PPARα-independent mechanisms, suggesting differences observed between PPARα activators may be attributed to drug-specific effects on non-PPARα targets [125]. The use of human-based organoid culture models and non-rodent animal models of HCC may help elucidate the antitumorigenic potential of PPARα activators. New more potent and selective drugs such as permafibrate, a SPPARMα, may bring new treatment options to NAFLD and NAFLD-related HCC.

#### 5.1.3. PPARδ

PPARδ is the least characterized of the PPARs. PPARδ is most highly expressed in intestinal and liver tissues [129,130,131], though its function in skeletal muscle has been the major focus [132,133,134]. Early studies demonstrated PPARδ activation may improve the serum lipid profile, notably HDL and TGs, as well as insulin resistance and obesity [135,136,137]. Animal and human studies then found PPARδ activation may also improve NAFLD [75,76,135,138,139,140,141,142,143]. Despite strong evidence for PPARδ activation in the treatment of metabolic disorders, a major setback occurred after the PPARδ agonist GW501506 was found to promote carcinogenesis, particularly colon cancer, in rodent models [135,136,144]. However, subsequent analyses and reviews have found PPARδ activation may indeed harbor antitumorigenic properties, though a consensus has not been reached and further investigation is needed [136,145]. Interestingly, the activation of PPARδ leads to hepatic accumulation of 16:0/18:1-phosphatidylcholine, a ligand of PPARα, which may confound studies utilizing rodent models of HCC as discussed above [146].

Elafibranor, or GFT505, is a dual PPARα/δ agonist that showed promise in early clinical trials, leading to improved serum lipid profiles and insulin resistance in dyslipidemic and prediabetic/diabetic patients, respectively [147,148]. These effects appeared to be mediated via hepatic PPAR signaling pathway activation rather than in skeletal muscle by gene expression analysis [148]. A mouse model of NASH also demonstrated elafibranor’s positive effects on histological and serum markers of disease [149]. Afterwards, a larger randomized control trial in approximately 300 patients with NASH without cirrhosis was performed comparing two doses of elafibranor to placebo, with the primary endpoint of NASH resolution without fibrosis worsening [150]. While the predefined endpoint was not met when using an older definition of NASH, the use of a newer modified definition showed the higher-dose (120 mg/day) elafibranor-treated group was more likely to meet primary endpoint when compared to placebo. These patients additionally displayed improved serum lipid profiles, insulin sensitivity, and markers of systemic inflammation. Unfortunately, in another major setback, a recent phase 3 trial (NCT02704403) found 72 weeks of elafibranor (120 mg/day) treatment in NASH fibrosis patients did not meet primary or secondary endpoints, though was confounded by a high rate of improvement in the placebo group. With preclinical models continuing to show NASH improvement with elafibranor treatment [151,152] as well as the suppression of HCC with PPARδ activation [153,154], the future of PPARδ-targeting therapies in the treatment of NAFLD and NAFLD-related HCC is possible.

### 5.2. Bile Acids

Primary bile acids are amphipathic molecules synthesized by hepatocytes from cholesterol [155]. They are conjugated and transported to bile canaliculi and subsequently the intestine where microbes may perform additional modifications, and then are either secreted in stool or, much more commonly, recycled back to the liver via the enterohepatic circulation [155]. Liver and plasma levels correlate with liver disease, but the composition of the bile acid pool is likely the biggest determinate of liver disease progression [155,156,157]. The relationship of bile acids to metabolic disorders, notably diabetes and NAFLD, has gained much interest in the past decade [155,158]. While bile acid accumulation in NAFLD may directly result in liver toxicity to promote cholestatic liver injury [159,160,161], specific bile acids also play major roles in cell signaling pathways. The most prominent example of the latter is the ability of the endogenous bile acid, chenodeoxycholic acid, to activate the major suppressor of hepatic bile acid synthesis, FXR [162]. Additionally, hepatic FXR activation promotes fatty acid beta oxidation and the suppression of lipogenesis, making it an especially attractive target in NAFLD [155,158,163]. Preclinical animal models found FXR activation improves NASH, insulin resistance, and the serum lipid profile [164,165,166]. In addition to their regulation of metabolic homeostasis, bile acids and their signaling pathways play important roles in carcinogenesis [167]. FXR knockout mice show increased levels of hepatic inflammation, notably through NF-κB activation [168], and display higher rates of HCC development [169,170,171]. The FXR agonist, GW4064, was found to suppress HCC in cell culture and a xenograft model through alterations in suppressor of cytokine signaling 3 (SOCS3), a major repressor of the signal transducer and activator of transcription 3 (STAT3) pathway [172]. FXR is downregulated in human HCC, and FXR knockout mice display liver transcriptome alterations consistent with human pathology [173].

The semi-synthetic bile acid analogue 6α-ethyl-chenodeoxycholic acid, more commonly known as obeticholic acid, was developed and is the first FXR agonist to be used in humans. In a co-culture of hepatocytes and stellate cells, obeticholic acid reduced collagen deposition in a dose-dependent manner [174]. Rodent models of metabolic syndrome found obeticholic acid reduced liver steatosis and fibrosis and insulin resistance [152,175,176,177]. A phase 2 randomized control trial found obeticholic acid improved insulin resistance and decreased markers of liver inflammation and fibrosis in diabetic NAFLD patients [178]. A subsequent clinical trial of 283 NASH patients in the United States demonstrated significantly improved liver histology with obeticholic acid compared to placebo (45% vs. 21%) [179]. Currently, a phase 3 multicenter randomized control trial (NCT02548351) of over 2000 NASH patients is underway, with an estimated completion date of October, 2022. An interim analysis of 931 patients showed an improvement in liver fibrosis scores with obeticholic acid treatment [180]. The most common adverse effect in these studies was pruritis, and increased rates of gallstone formation have been noted elsewhere [181]. Obeticholic acid has also been shown to have antitumorigenic properties against HCC, with a recent study showing alterations in interleukin-6 (IL-6)/STAT3 signaling [182]. While obeticholic acid is FDA approved for the treatment of primary biliary cholangitis, it was recently denied approval for the treatment of NAFLD and sent back to the pharmaceutical manufacturer (Intercept) with request for further efficacy data and safety analysis. Obeticholic acid or other FXR agonists (ex. Px-104) and bile acid analogues (ex. arachidyl amido cholanoic acid, i.e., Aramchol) have potential in the future of NAFLD treatment and NAFLD-related HCC chemoprevention.

Bile acid metabolism is complex, with numerous enzymes, signaling molecules, receptors, and transporters working in tandem. A notable illustration of this is the intestinal expression of FXR to sense bile acid concentrations, which upon activation leads to the increase in circulating fibroblast growth factor (FGF) 19 (FGF15 in mice) [183]. FGF15/19 binds to hepatocyte fibroblast growth factor receptor 4 (FGFR4) to inhibit cholesterol 7α-hydroxylase A1 (CYP7A1), the rate-limiting enzyme in bile acid synthesis [183]. Additionally, while bile acids appear in far higher concentrations in the intestine and liver, they also play a direct role in systemic lipid and glucose metabolism and inflammation through various receptors [155,158]. The use of drugs and drug combinations targeting various bile acid-signaling molecules (FGF15/19), bile acid receptors (G protein-coupled bile acid receptor 1 (TGR5) and FGFR4), and bile acid transporters (apical sodium-dependent bile acid transporter (ASBT)) may provide novel targets in the approach to NAFLD and liver cancer [155,158,163,184].

### 5.3. GLP-1 Receptor Agonists

Glucagon-like peptide-1 (GLP-1) receptor agonists (GLP-1RAs) such as liraglutide and exenatide are predominantly used for their antihyperglycemic properties. Upon feeding, the incretin GLP-1 is released by enteroendocrine L cells where it binds to GLP-1 receptors on pancreatic beta cells [185,186]. GLP-1 receptor binding promotes glucose-dependent pancreatic beta cell insulin release improving hyperglycemia as well as beta cell health to prevent diabetes progression. GLP-1 receptors may also be expressed on hepatocytes to modulate insulin signaling and lipid metabolism [187], though this has been debated and liver effects may be indirect [188,189]. However, a number of studies have reported their presence in human hepatocytes and have demonstrated direct effects of GLP-1RAs on hepatocytes and HCC cell lines in vitro [187,190,191,192,193,194]. Preclinical animal models have also demonstrated their positive effects on liver lipid metabolism and NAFLD [190,195,196,197]. Responsible mechanisms found include increased AMPK and PPARα signaling along with modulation of SREBP1c and stearoyl-CoA desaturase-1 (SCD1) expression. A recent systematic review of 24 completed and 8 ongoing clinical trials found strong evidence for the use of GLP-1RAs in the treatment of NAFLD patients [198]. Shifting focus to HCC, preclinical animal models demonstrate the antitumorigenic properties of GLP-1RAs [194,199]. The corresponding antitumorigenic effect in vitro using cell culture models support a direct and independent mechanism [192,193,194]. Dipeptidyl peptidase-4 (DPP-4) inhibitors are antidiabetic agents that enhance circulating levels of incretins such as GLP-1. These agents are also under investigation in the treatment of NAFLD and have been found to harbor direct antihepatocarcinogenic properties in rodent models [200].

### 5.4. ACC Inhibitors

While increased hepatic de novo lipogenesis is a primary driver of the pathogenesis of NAFLD, it also plays a major role in HCC [201,202]. AMPK, a master energy sensor of the cell, has long been recognized in metabolic disorders and was also found to be a tumor suppressor in HCC [203,204]. In instances of nutrient deprivation and accumulation of cellular AMP, AMPK becomes activated to perform inhibitory phosphorylation of acetyl-CoA carboxylase (ACC). In hepatocytes, the two isoforms ACC1 and ACC2 are found in the cytosol and mitochondria, respectively, and function to convert acetyl-coenzyme A (acetyl-CoA) to malonyl-CoA, the rate-limiting step of de novo lipogenesis [205]. Malonyl-CoA inhibits carnitine palmitoyltransferase 1 (CPT1) to suppress fatty acid transport for mitochondrial beta oxidation. Therefore, in theory, ACC inhibition should decrease hepatic lipogenesis and promote fatty acid oxidation to blunt NAFLD progression. ACC inhibitors were found to reduce liver steatosis and insulin resistance in rodent models, though may worsen dyslipidemia [206,207,208]. Firsocostat (GS-0976), a dual ACC1/ACC2 inhibitor, reduced liver steatosis and serum markers of fibrosis in an early clinical trial [209,210], though unfortunately a recent phase 2 trial did not show similar efficacy and meet the study’s primary endpoint of fibrosis improvement (NCT03449446). Other ACC inhibitors are also being evaluated in clinical trials and have shown to reduce hepatic de novo lipogenesis [211]. In addition to reducing liver steatosis, ACC inhibition has also been shown to play an independent therapeutic role in HCC [212]. ACC and other targets of hepatic lipogenesis and fatty acid oxidation could modulate lipid metabolism to treat NAFLD and confer additional antitumorigenic effects.

### 5.5. Statins

The rate-limiting step of de novo cholesterol synthesis is carried out by 3-hydroxy-3-methylglutaryl-coenzyme A reductase (HMGR) [213]. Statins inhibit HMGR and are most known for their role in the treatment of dyslipidemia which confers primary and secondary prevention of cardiovascular disease. The high rates of dyslipidemia and statin use have allowed for powerful retrospective analyses. In regard to NAFLD, systematic reviews and meta-analyses have demonstrated a possible decrease in serum aminotransferase levels and steatosis on ultrasound though histological results were limited and conflicting [214,215,216]. However, numerous reviews focused on statins in the context of HCC have consistently demonstrated a negative correlation between statin use and tumor formation in liver disease of various etiology [217,218,219,220,221,222,223]. Additionally, in vitro studies and preclinical animal models have demonstrated this antitumorigenic effect with c-myc and IL-6/STAT3 pathways implicated [224,225,226]. Overall, there appears to be evidence for potential statin-mediated independent antitumorigenic effects in HCC making them suitable agents for chemoprevention or even adjuvant therapy, though these epidemiologic data are limited by their retrospective nature and prospective randomized trials are needed. While statins are associated with liver enzyme elevations, they have been shown to be safe in NAFLD patients without decompensated cirrhosis [227]. NAFLD patients often present with concomitant dyslipidemia in which case statin therapy is recommended. The use of statin therapy may reduce HCC risk in non-dyslipidemic NAFLD patients and serve a role in chemoprevention, though it is not currently indicated. While it will not be discussed in detail, metformin similarly has not consistently prevented NAFLD progression though strong epidemiologic data and preclinical models show its chemopreventive effects in HCC through alterations in AMPK and ACC pathways.

## 6. Future Perspectives

### 6.1. Gut Microbiome

Knowledge of the gut microbiome’s relationship to human health and disease has rapidly advanced over the last decade [228,229]. These organisms populate the gastrointestinal tract, where they alter dietary food substrates and secrete a host of effector metabolites. Microbes affect host physiology directly through cell membrane components ex. endotoxins/lipopolysaccharides, and indirectly through metabolite mediators ex. short-chain fatty acids and secondary bile acids. This rapidly growing field of study has led to discoveries related to cardiovascular disease, inflammatory bowel disease, and liver disease, among others [228,230]. The anatomic location of the liver links it closely as an effector organ of gut microbe physiology as gastrointestinal metabolites primarily drain into the portal venous circulation for initial delivery to the liver prior to entrance into the systemic circulation. This design places the liver in prime position to serve as a biotransformation factory of these molecules and there is now strong interest of the gut microbiome’s relationship to both NAFLD and HCC [230,231].

Strong associations between the gut microbiome and NAFLD have been made [230]. Alterations in the gut microbial composition (dysbiosis) and gut membrane integrity have been strongly linked to liver disease, especially in patients with late-stage fibrosis and cirrhosis. Dysbiosis leads to dysregulation of bile acid homeostasis, local and systemic inflammation, and the release of pathogen- and microbial-associated molecular patterns (PAMPs/MAMPs) and microbial metabolites. Compromised gut membrane integrity exaggerates these effects. Diagnostic and therapeutic options are now being considered [232]. Gut microbe composition may serve as a useful tool for non-invasive diagnosis and severity assessment of NAFLD. Correcting dysbiosis through dietary interventions and pro-, pre-, and synbiotics are under active investigation as well as the use of postbiotics, antibiotics, fecal microbiota transplantation, and bacteriophage therapy. Methods to reduce NAFLD-promoting pathways that arise from exposure to harmful microbial components and metabolites are being explored. These include improving gut membrane integrity, targeting liver physiology such as through toll-like receptor (TLR) inhibition, and even altering bacterial gene pathways to improve the microbial-produced metabolome. A prime example of this last point is the investigation of choline trimethylamine (TMA)-lyase (CutC) inhibitors to reduce bacterial generation of the harmful microbial-metabolite TMA [233].

The association of dysbiosis and altered gut membrane integrity in HCC has been established [231], though may be confounded by the impact of coexisting cirrhosis. The microbiome appears to play an independent role in hepatocarcinogenesis in addition to promoting liver injury and NAFLD as discussed above, though studies have been primarily done in animal models and are limited by a lack of human clinical data. Rodent models demonstrate the contribution of the gut microbiome in tumor development, as germ-free and microbiome-ablated animals are protected from HCC development [231]. In line with this, treatment with MAMPs and compounds that impair gut membrane permeability lead to increased tumor burden. Pro-tumorigenic mechanisms implicated include the activation of hepatic TLRs by bacterial endotoxin, altered bile acid metabolism with increased levels of deoxycholic acid, production of hepatotoxic metabolites such as TMA, and alterations in immune cell activation and immunosurveillance [231,234]. Future studies are needed to better delineate mechanistic pathways and identify therapeutic opportunities.

### 6.2. Lipotoxicity and Autophagy

**Figure 2:**
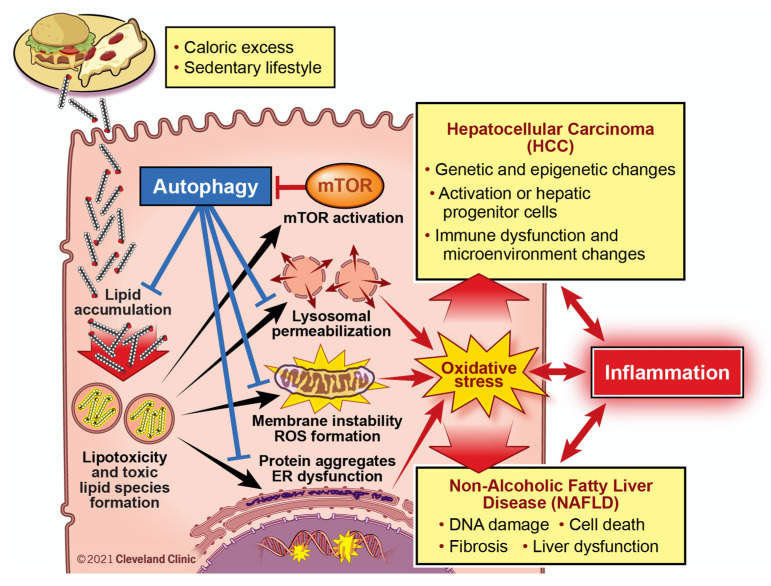
Targeting Lipotoxicity-Driven Pathways to Reduce NAFLD Progression and Prevent HCC Development. The initiating event in NAFLD is lipid accumulation, and it is generally accepted that “lipotoxicity” occurs where intermediates in lipid biosynthesis can act as signaling molecules or alter other cellular processes to promote liver disease. Lipotoxicity is associated with many cellular pathways including increased oxidative stress, endoplasmic reticulum (ER) stress, mitochondrial dysfunction with reactive oxygen species (ROS) formation, and lysosomal dysfunction with lysosomal membrane permeabilization (LMP). Collectively, the pathways initiated by lipotoxicity may lead to NAFLD progression via DNA damage and cell death, inflammatory cell recruitment, activation of pro-fibrotic programs, as well as local and systemic insults. Autophagy is central to several of these lipotoxicity-driven pathways, and therapies impacting autophagic flux during NAFLD progression may hold promise.

An abundance of literature exists on the pathogenesis of NAFLD and of HCC, though usually independent from one another. Interest of overlapping pathogenic mechanisms has grown due to the rise in NAFLD-related HCC and the recognition of metabolic syndrome’s negative effects on HCC of various etiology. A review on this was written by Anstee et al. and emphasized the importance of oxidative stress, DNA damage, and autophagy in NAFLD progression and HCC development [235]. An excellent review series on lipotoxicity-mediated cell dysfunction nicely illustrates pathogenic mechanisms in the development and progression of NAFLD that closely relate to hepatocarcinogenesis [236,237,238,239,240]. These will serve as the basis of Figure 2 and the following paragraphs to highlight an untapped potential in the approach to NAFLD and its progression to HCC.

NAFLD is characterized by liver steatosis, the accumulation of fat deposition within the liver. Hepatic free fatty acid accumulation generates a host of toxic lipid intermediates to trigger various forms of cell stress [237,238]. This phenomenon known as “lipotoxicity” is ultimately the driver of NAFLD and should be maintained as a major focus in targeting pathogenic mechanisms. Lipotoxicity involves the accumulation of α,β polyunsaturated lipid aldehydes and other toxic lipid intermediates such as palmitate and lysophosphatidylcholine, leading to endoplasmic reticulum stress, mitochondrial dysfunction, and lysosomal stress. These culminate in oxidative stress, which itself can exacerbate cell dysfunction creating a vicious positive feedback loop. Oxidative stress in NAFLD and its relationship with hepatocyte injury, immune system activation, and collagen deposition by hepatic stellate cells has been nicely reviewed elsewhere [241,242,243,244]. These insults promote NAFLD progression through processes involving DNA damage, cell death, local and systemic inflammation, fibrosis, and liver dysfunction [8,235]. These work together to promote HCC development and progression via the activation of oncogenes and inhibition of tumor suppressor genes, aberrant cell cycle regulation, activation of hepatic progenitor cells, and immune dysfunction along with a tumor-promoting microenvironment [235,245,246,247].

Autophagy is known for its “self-eating” functions serving to maintain cellular homeostasis through organelle recycling, especially during periods of starvation [239]. Autophagy and lysosomal pathways function in various ways to limit the lipotoxicity-mediated pathways listed above [239]. Lipophagy, a subset of autophagy, works to reduce lipid accumulation when canonical lipid catabolic pathways are overwhelmed. Additionally, autophagy in concert with lysosomes promotes lipid turnover to prevent accumulation of toxic lipid intermediates. If ER stress pathways and the unfolded protein response are unable to maintain cellular protein homeostasis, autophagy is stimulated to degrade misfolded and aggregated proteins. Mitochondrial and lysosomal health are maintained by autophagy through mitophagy and lysosomal membrane turnover. Lastly, autophagy functions as a critical component of the cellular antioxidant response to reduce ROS formation and cell damage [248], and by doing so also prevents various mechanisms linked to hepatocarcinogenesis [249]. As such, autophagy promotes cellular health in the face of lipid overload and is an attractive target in the treatment of NAFLD. Additionally, it plays critical roles in cellular differentiation [250], cell cycle regulation [251], and the modulation of tumor microenvironments [252], and has been implicated in numerous cancers including HCC though its role in treatment is unclear [249,253].

The mammalian target of rapamycin (mTOR), a master nutrient sensor of the cell, is activated by excess free fatty acid and carbohydrate and acts as a repressor of autophagy [254]. mTOR activation has been strongly linked to both NAFLD and HCC. In the latter, mTOR was found to be upregulated in approximately 50% of human tumors and associated with poorer prognosis [254], with one study implicating it directly promotes tumorigenesis through lipid synthesis [255]. Inhibitors of mTOR (rapamycin and analogs) are being used in the treatment of other cancers and are being investigated in the treatment of HCC though have not shown a clear benefit [256]. No prospective studies have been done to discern their chemopreventive potential. As autophagy promotes cellular health under normal conditions and periods of starvation, there is concern it may act as a double-edged sword to promote tumor cell health and cancer progression. Overall, however, this notion is not well supported by in vitro cell culture models, preclinical animal models, or human data.

With the exception of the antioxidant vitamin E, the lipotoxicity-mediated pathways outlined above (Figure 2) have remained largely untapped in the treatment of NAFLD and may serve as powerful adjuncts in the chemoprevention of NAFLD-related HCC. A precise therapeutic induction of autophagy or modulation of related endocytic and lysosomal pathways may serve to modulate several pathogenic mechanisms simultaneously. New approaches to harness the beneficial effects of autophagy on liver disease are being considered. Studies using intermittent fasting (NCT04355910) and second-generation mTOR inhibitors are especially exciting [256,257]. New therapeutic options may open in the form of monotherapy or in combination with other treatments. A better understanding of these mechanisms along with their complex interrelationships is needed.

## 7. Conclusions

Rates of metabolic syndrome and NAFLD continue to rise globally. Although HCC was classically thought of as a disease initiated by viral hepatitis, effective antiviral therapies are now available and NAFLD continues to gain ground as a leading cause of chronic liver disease and HCC. The treatment of NAFLD has remained largely unchanged and it is expected that rates of NAFLD-related HCC will continue to sharply rise. New treatment approaches are needed. The current paradigm of NAFLD treatment focuses on improving markers of liver injury and fibrosis, though the prevention of HCC should also be considered. Pathogenic mechanisms common to both NAFLD and HCC may also serve as targets for chemoprevention. Metabolic syndrome promotes tumor formation and progression in non-NAFLD-related HCC, and a similar chemopreventive approach in these patients targeting metabolically relevant pathways may prove beneficial.

The pathogenesis of NAFLD encompasses aberrant lipid and nutrient homeostasis within a complex web of cellular pathways. These intricate pathways working in tandem to achieve a delicate system of checks and balances may be responsible for the failure rates that have plagued NAFLD clinical trials. Combination therapy to simultaneously manipulate these mechanisms of disease may help to overcome this pharmacologic challenge and increase chances of therapeutic success. While awaiting more effective NAFLD treatments, adopting a strategy of primary prevention against HCC in these patients may be useful. Several therapeutic targets have demonstrated potential in reducing HCC development in NAFLD and prospective trials are needed. A better understanding of pathways linking the progression of NAFLD to tumorigenesis may be used to develop treatment strategies to mitigate the development of HCC and its complications in these patients.
